# Peripheral Blood Genetic Biomarkers for the Early Diagnosis of Hepatocellular Carcinoma

**DOI:** 10.3389/fonc.2021.583714

**Published:** 2021-03-11

**Authors:** Ting Song, Li Li, Shaobo Wu, Yan Liu, Caiping Guo, Wen Wang, Lili Dai, Tong Zhang, Hao Wu, Bin Su

**Affiliations:** ^1^Department of Infectious Diseases and Medical Immunology, Beijing Youan Hospital, Capital Medical University, Beijing, China; ^2^Beijing Key Laboratory for HIV/AIDS Research, Beijing, China; ^3^Department of Hepatology, The Sixth People's Hospital of Qingdao, Qingdao, China; ^4^Center of Transfusion-Transmitted Infectious Diseases, Institute of Blood Transfusion, Chinese Academy of Medical Sciences (CAMS), Chengdu, China

**Keywords:** hepatocellular carcinoma, biomarkers, microRNA, exosome, nucleic acid

## Abstract

Hepatocellular carcinoma (HCC) is one of the most common cancers worldwide and has high mortality. Biomarkers related to HCC, such as alpha-fetoprotein, and imaging technology, such as ultrasound and computed tomography, have been used to screen and monitor HCC, but HCC is still difficult to diagnose effectively in the early stage due to the low sensitivity of the above mentioned traditional methods. There is an urgent need for noninvasive biomarkers to facilitate the screening and early diagnosis of HCC. With the advancement of next-generation sequencing, genetic biomarkers are becoming the core of cancer diagnosis. Genetic biomarkers such as peripheral blood circulating tumor DNA, microRNAs, long noncoding RNAs, circular RNAs, and exosomes have become the focus of early HCC diagnostics. HCC genetic biomarkers have been implemented in clinical practice. In this review, we describe the available literature on peripheral blood genetic biomarkers in the diagnosis of early HCC.

## Introduction

**Figure 1:**
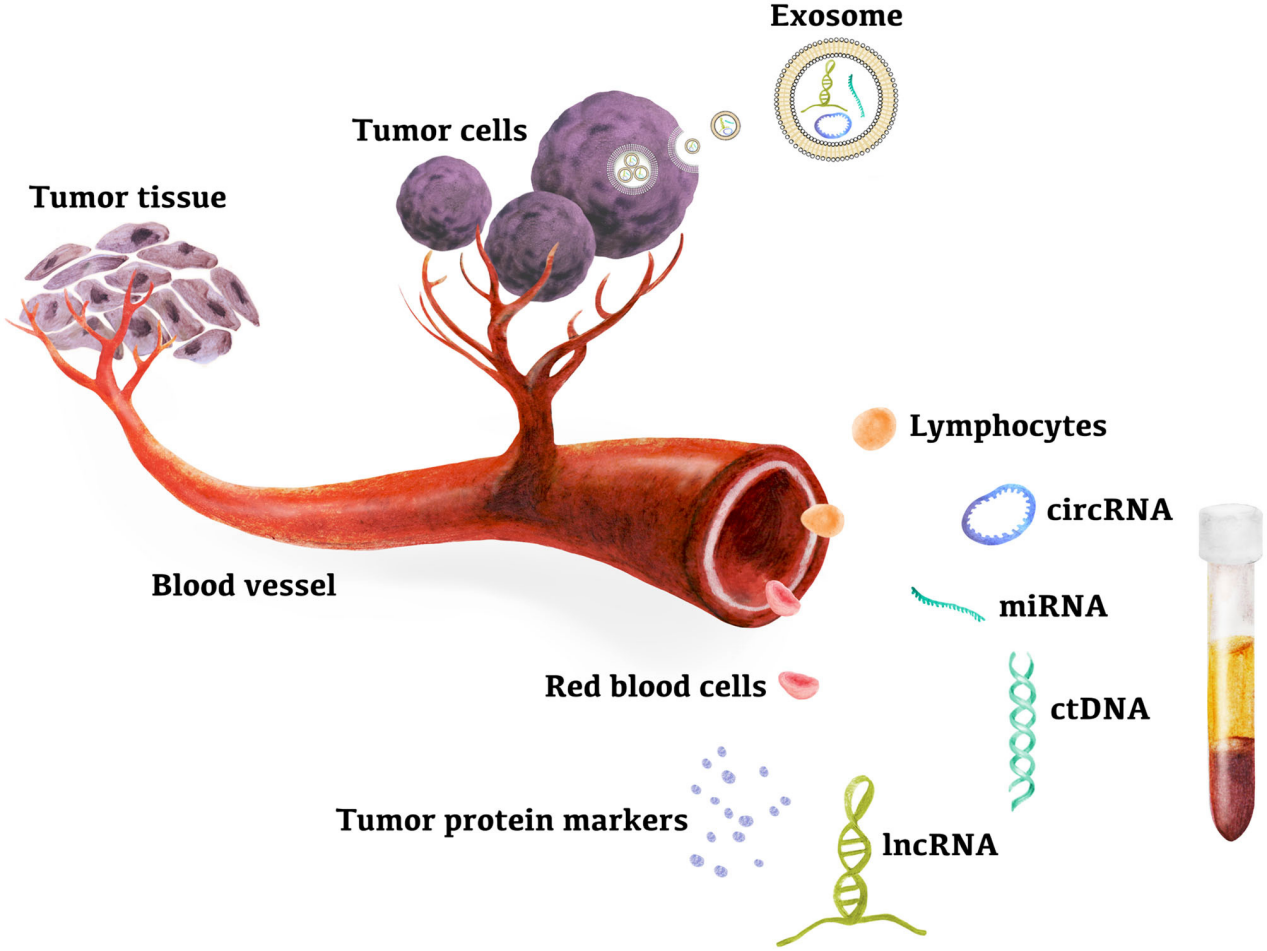
Peripheral blood genetic biomarkers for HCC early diagnosis. Evidences suggested that ctDNAs in plasma and miRNAs, lncRNAs, circRNAs in exsome, plasma, and PBMC are new candidates for high sensitive biomarkers for early diagnosis of hepatocellular carcinoma. miRNA, microRNA; lncRNA, long noncoding RNA; circRNA, circular RNA; ctDNA, circulating tumor DNA.

Liver cancer is the fifth most common cancer worldwide and has become the third leading cause of cancer-related deaths, based on reports from the World Health Organization. Hepatocellular carcinoma (HCC) accounts for ~90% of primary liver cancer, and chronic hepatitis B virus (HBV) and hepatitis C virus (HCV) infection is the main cause of HCC, accounting for 80% of HCC cases worldwide (1–3). Approximately 15% of HCC cases are attributed to nonalcoholic fatty liver disease, alcoholic cirrhosis, and other causes (4, 5). The annual incidence of HCC in patients with liver cirrhosis (LC) by any cause is 2–4% (5, 6). Early diagnosis of HCC is difficult because most patients with cirrhosis are asymptomatic. Therefore, over the past few decades, deaths related to HCC have increased significantly. Most HCC patients are found to be in an advanced stage and face 1-year survival rates of 15–39% with limited treatment options (7, 8). The overall 5-year survival rate for HCC patients is 10–20% (9). However, early diagnosis can significantly improve HCC prognosis, and emerging evidence has shown that surgical resection and liver transplantation in patients with early detection may be the best chance for the treatment of HCC (10). The 5-year survival rate of patients with early HCC treated by hepatectomy or liver transplantation increased to 60–70% (11–14). Moreover, a meta-analysis of 47 studies involving 15,158 patients showed that overall survival improved by diagnosing HCC at a very early stage, meaning that patients were eligible for potentially curative therapies such as surgical resection and liver transplantation (15). Clinical practice guidelines for HCC recommend the use of imaging tests such as computed tomography (CT), magnetic resonance imaging (MRI), ultrasound (US), or a combination with serum alpha-fetoprotein (AFP) assay for the diagnosis of HCC at an early stage (16). However, imaging has limitations in diagnostic accuracy and sensitivity, while common serum markers show poor diagnostic performance (5). Histological biopsy is the gold standard for the diagnosis of HCC, and the risks of biopsy and the difficulty to take a biopsy sample at the early tumor stage have limited its wide applicability. Therefore, it is urgent to find new biomarkers with high sensitivity and specificity that are useful in the early diagnosis of HCC. With the advent of high-throughput sequencing technology, genetic biomarkers such as cell-free DNA (cfDNA), epigenetic changes, and circulating RNA [microRNAs (miRNAs), long noncoding RNAs (lncRNA), and circular RNAs (circRNA)] from peripheral blood have become the focus of the early diagnosis of HCC. HCC genetic biomarkers have been widely developed and integrated in clinical practice, as shown in Figure 1. In this review, we aimed to describe current studies on the detection of miRNAs, lncRNAs, circRNAs, exosomes, and circulating tumor DNA (ctDNA) in peripheral blood as candidate genetic markers for the early diagnosis of HCC. Eventually, early diagnosis will provide more treatment options and improve the survival rate of HCC patients.

## MicroRNAs

MiRNA is a short single-stranded noncoding endogenous RNA molecule with a length of ~22 nucleotides. It regulates gene expression by targeting messenger RNA (mRNA), leading to degradation or inhibition of translation (17, 18). MiRNAs play an important role in cell development, proliferation, and apoptosis, and they may regulate cell death resistance and avoid immune damage, tissue invasion/metastasis, angiogenesis, and gene mutation, contributing to tumorigenesis and cancer progression (17). MiRNAs in serum and plasma have been identified as tumor biomarker targets. There is a correlation between abnormal circulating miRNA expression levels and the clinicopathological characteristics of certain malignancies (such as breast cancer, prostate cancer, lung cancer, and HCC) (19–22). Circulating miRNAs are stable, readily available and not affected by RNase activity in the pathogenesis of HCC; thus, circulating miRNAs are the promising early-stage HCC biomarkers (22, 23). Numerous studies have been conducted on HCC biomarkers of circulating miRNAs (as shown in Table 1).

**Table 1:** Sensitivity and specificity of selected studies and blood biomarkers for detecting hepatocellular carcinoma.

**miRNA**	**Study sample**	**Sensitivity (%)**	**Specificity (%)**	**Publication date**	**References**
miR-21	515 HBV-HCC vs.89 CHB or 249 HC	73.3–88.4	68.4–87.0	2019	(24, 25)
miR-15b	57 HBV-HCC vs.59 (HC+CHB)	98.30	15.30	2012	(26)
miR-18a	86 HBV-HCC vs.45 HC	86.10	75.00	2012	(27)
	86 HBV-HCC vs. 30 (LC+CHB)	77.20	70.00	2012	(27)
miR-26a	52 HCC vs. 43 HC	51.90	95.20	2016	(28)
	52 HCC vs. 42 CH	75	70	2016	(28)
	25 HCC vs. 42 CH	80	62.50	2016	(28)
miR-101	52 HCC vs. 43 HC	47.10	81	2016	(28)
	52 HCC vs. 42 CH	54.90	76.90	2016	(28)
miR-125b	64 HBV-HCC vs. 63 CHB	100	75.50	2017	(29)
MiR-129	36 HCC vs. 36 HC	100	97.20	2019	(30)
miR-130b	57 HCC vs. 30 HC	87.70	81.40	2012	(26)
miR-122	40 HCV-HCC vs. 40 CHC	87.50	97.50	2017	(31)
	40 HCV-HCC vs.20 HC	87.50	95	2017	(31)
	85 HCC vs. 85 HC	70.60	67.10	2013	(32)
	38 HCV-HCC vs. 42 CHC	85.71	83.33	2019	(33)
miR-375	120 HBV-HCC vs. 210 HC	96.00	100.00	2010	(34)
miRNA125a	38 HCV-HCCvs.42 CHC	82.35	83.33	2019	(33)
miRNA139	38 HCV-HCC vs. 42 CHC	85.71	69.44	2019	(33)
miRNA145	38 HCV-HCC vs. 42 CHC	88.64	75.76	2019	(33)
miRNA199a	38 HCV-HCC vs. 42 CHC	87.80	65.79	2019	(33)
miR-224	40 HCV-HCC vs. 40 CHC	87.50	97	2017	(31)
	40 HCV-HCC vs. 20 HC	92.50	90	2017	(31)
	1,997 HCC vs. 1,851 BP	86.8	79.2	2020	(35)
miR-214	224 HCV-HCC vs. 250 LC	92.90	75.50	2017	(36)
miR-1269	224 HCV-HCC vs. 250 LC	78.60	59.80	2017	(36)
miR-23a	57 HCC vs. 57 LC	89.47	64.91	2017	(37)
miR-375	40 HBV-HCC vs. 137 CHB	73.9	93	2020	(38)
miR-372	40 HCV-HCC vs. 20 HC	90.6	100	2020	(39)
miR-215	60 HCC vs. 75 LC	78.3	88	2019	(40)
miR-4651	366 HCC vs. 662 HC	70	90	2017	(29, 41)
miRNA-27a	51 HCV-HCC vs. 39 LC	96.7	71.7	2018	(42)
miRNA-18b	51 HCV-HCC vs. 39 LC	75.6	46.6	2018	(42)
miR-122 + AFP	40 HCV-HCC vs. 40 CHC	97.50	100	2017	(31)
miR-224 + AFP	40 HCV-HCC vs. 40 CHC	90	100	2017	(31)
miR-27b and miR-192	212 HBV-HCC vs. 110 HC	95.2	68.5	2017	(43)
	212 HBV-HCC vs. 106 LC	79.3	78.5	2017	(43)
miRNA-27a and miRNA-18b	40 HCV-HCC vs.40 LC	91.1	71.7	2018	(42)
miR-20a, miR-320a, miR-324 and miR-375	35 HBV-HCC vs. 50 CHB	65.0	77.5	2015	(44)
	32 HBV-HCC vs. 32 CHB	56.0	83.8	2015	(44)
miR-21, miR-26a, miR-101 + AFP	52 HCC vs. 43 HC	87.0	78.0	2016	(28)
miR-15b and 130b	57 HBV-HCC vs.59 (HC+CHB)	98.2	91.5	2012	(26)
miR-375, 25 and let-7f	120 HBV-HCC vs. 210 HC	100.0	96.0	2010	(34)
miR-122, 192, 21, 223, 26a, 27a and 801	196 HCC vs. 66 HC	93.9	83.2	2011	(45)
miR-375, 23b, 423, 23a and 342	120 HBV-HCC vs. 210 HC	96.9	99.4	2010	(34)
miR-10a and 125b	120 HBV-HCC vs. 135 (CHB + LCB)	98.5	98.5	2010	(34)
miR-206, 141, 433, 1228, 199a, 122, 192 and 26a	261 HBV-HCC vs. 173 HC	82.3	83.3	2014	(46)
	261 HBV-HCC vs. 233 LC	81.6	84.6	2014	(46)

*CHC, chronic hepatitis C infection; CHB, chronic HBV hepatitis; HBV-HCC, HCC related with HBV infection; HCC, hepatocellular carcinoma; HC, healthy control; LCB, liver cirrhosis of hepatitis B; CH, chronic hepatitis; HCV-HCC, HCC related with HCV infection; LC, liver cirrhosis; BP, benign patients*.

A meta-analysis evaluated microRNA-21 as a biomarker for the early diagnosis of HCC. A total of 515 HCC patients and 338 healthy controls (HCs) or patients with chronic hepatitis (CH) were included in six published studies from different laboratories. The pooled sensitivity and specificity of miR-21 for HCC diagnosis were 85.2% (73.3–88.4%) and 79.2% (68.4–87.0%), respectively. The area under the curve-receiver operating characteristic curve (AUC-ROC) was 0.89, and it was concluded that miR-21 is a useful biomarker for the early diagnosis of HCC (24). However, further large-scale prospective studies are needed to validate the clinical application of miR-21 for the early diagnosis of HCC. Previous studies reported that the level of serum miR-21 in HCC patients was significantly higher than that in patients with chronic hepatitis (CH) and healthy controls (HCs). MiR-21 belongs to oncogenic miRNA and has oncogenic effects. MiR-21 targets tumor suppressor genes such as programmed cell death 4, phosphatase, and tensin homolog and promotes HCC cell proliferation, migration and invasion (47, 48). A study evaluated circulating miRNAs as biomarkers for the early diagnosis of HCC in Egypt and the results showed that miR-214 and miR-1269 could be used as early biomarkers for tracking the progression of LC to HCC with AUC values of 0.842 (sensitivity 92.9% and specificity 75.5%) and 0.691 (sensitivity 78.6% and specificity 59.8%), respectively. Further research found that the AUC value of the miRNA panel (miR-214, miR-125b, miR-1269, and miR-375) detected HCC in patients with liver cirrhosis (LC) increased to 0.99 with specificity 98.3% and sensitivity 98.7%. The miRNA panel represents an accurate and sensitive biomarker for the early diagnosis of HCC (36). Another study evaluated circulating miRNAs as biomarkers for the early screening of HCC in patients from China. In the exploration phase, high expression of miR-15b and miR-130b in HCC tumor tissues was identified. These miRNAs are also detectable in serum samples of HCC patients and are sharply reduced after tumor surgical resection, showing that miR-15b and miR-130b are from tumor-derived sources. In the validation phase, the sensitivity and specificity of miR-130b for detecting HCC from chronic hepatitis B (CHB) and HC were 87.7% and 81.4%, respectively. MiR-15b had the highest sensitivity (98.3%) but low specificity (15.3%) in detecting HCC. The sensitivity and specificity of combined miR-15b and miR-130b in detecting HCC improved to 98.2 and 91.5%, respectively, and the accuracy rate of combined miR-15b and miR-130b in the diagnosis of early-stage HCC was 97.8%. The results show that miR-15b and miR-130b may be used as biomarkers for early HCC detection (26). Tan et al. proposed and validated the serum miRNA panel (8 differentially expressed miRNAs: miR-206, miR-141, miR-433, miR-1228, miR-199a, miR-122, miR-192, and miR-26a) as biomarkers to detect HCC in training and validation cohorts. The sensitivity of the miRNA panel in detecting HCC in patients with LC was higher than that of AFP (cut-off value 20 ng/mL) (81.6 vs. 57.3%), while the specificity of the miRNA panel was similar to that of AFP (84.6 vs. 79.5%). The diagnostic performance of the miRNA panel in HCC was better than that of AFP (46). Other studies have shown that the AUC value of the miRNA panel (seven differentially expressed miRNAs: miR-29a, miR-29c, miR-133a, miR-143, miR-145, miR-192, and miR-505) is higher than AFP to distinguish patients with HCC from controls. The miRNA panel had a higher AUC than AFP in identifying small HCC (0.833 vs. 0.727) and early-stage HCC (0.824 vs. 0.754) and can also detect AFP-negative HCC with an AUC value of 0.825 (49). Previous studies reported that the levels of serum miR-192, miRNA-125b and miR-23a in HCC patients could serve as biomarkers for predicting microvascular invasion and survival time (48, 50–52). Recently, Amr et al. assessed the diagnostic potential of miR-122 and miR-224 as biomarkers for early HCC. The sensitivity of miR-122 and miR-224 distinguishing early HCC from CH is 87.5%, and the specificity of miR-122 and miR-224 is 97.5 and 97%, respectively. The diagnostic accuracy of miR-122 and miR-224 in distinguishing early HCC from CH is 0.98 and 0.93, respectively (31). Other studies from different labs have also evaluated the sensitivity and specificity of miR-122 to distinguish early HCC from the control group (32, 33). These studies indicate that the sensitivity to distinguish HCC from HC and CHC is 70.6 and 85.71%, and the specificity is 67.1 and 83.33%, respectively. Therefore, miR-122 and miR-224 can be used as markers for early diagnosis of HCC. It is worth noting that miR-26a can distinguish early-stage HCC from CH, with an AUC value of 0.753 (80% sensitivity and 62.5% specificity) (28). These results show that miRNAs may be a suitable target for the development of the next generation of biomarkers for early diagnostic HCC, and miRNA panels or combined miRNAs and proteins such as AFP may provide a high diagnostic accuracy of HCC as compared with single miRNA. However, these studies had some limitations, since the enrolled patients mainly originated from Asia, Europe and America, and fewer African populations were enrolled. Therefore, the expression of miRNAs in HCC patients was not explored in different ethnicities. At present, there is no consensus on which miRNAs or combinations of miRNAs are more suitable for early HCC diagnosis. The profiles of miRNA expression in different etiologies of HCC (HBV-HCC, HCV-HCC, and nonalcoholic fatty liver disease-associated HCC) are also different, and further research is needed. The upregulation of some miRNAs was detected in patients with chronic liver inflammation. Thus, these miRNAs must be selected for combination with other markers for HCC diagnosis.

The miRNA-based targeted therapy of HCC may be a potential strategy for anticancer immunotherapies. Recently, miRNAs such as miR-16, miR-21, miR-155, and miR-34 have been introduced as therapeutic targets in clinical trials for cancer treatment, including multiple solid tumors and hematological malignances, and have improved the overall survival rate of cancer patients (53, 54). A miRNA-based strategy may be suitable for the development of the next generation of HCC treatments.

## Long Noncoding RNAs

MiRNA is not the only nucleic acid used as a biomarker for HCC, but the long noncoding RNA (lncRNA) is also a current research hotspot as a potential biomarker (55). The lncRNAs are a subgroup of noncoding RNA transcripts over 200 nucleotides in length and play a key role in the onset and development of cancers. They have been recognized as emerging stars in tumor research due to their important functions in cancer biology (56–58). Growing evidence has confirmed that many lncRNAs are frequently dysregulated in human cancers and participate in various biological processes, such as cell proliferation, apoptosis, differentiation, angiogenesis, invasion, and metastasis (59–65). The expression and dysregulation of many lncRNAs exhibit cell type specificity and cancer type specificity (66–68). Recently, evidence has shown that abnormal lncRNA expression is related to the initiation and progression of HCC (67, 69–71). Therefore, lncRNAs may be ideal HCC biomarkers and have potential use in the diagnosis of early HCC. LncRNAs were evaluated as candidate biomarkers for HCC diagnosis, as summarized in Table 2.

**Table 2:** Application of lncRNAs as a diagnostic index in hepatocellular carcinoma patients.

**LncRNAs**	**Study sample**	**Sensitivity (%)**	**Specificity (%)**	**References**
DANCR	HCC vs. LC + CHB	80.80	84.30	(72)
	HCC vs. LC + CHB +HC	80.80	84.30	(72)
LNC NEAT	HCC vs. HC	100	88.90	(30)
MALAT1	HCC vs. CH +HC	51.10	89.30	(73)
JPX	HCC vs. HC	100.00	52.40	(74)
c-JUN	HCC vs. HC+ CHC	91.40	91.40	(75)
	Early HCC vs. HC	93.10	91.40	(75)
SNHG1	HBV-HCC vs. LC+ CHB	70.10	68.20	(76)
	HBV-HCC vs. HC	87.30	86.00	(76)
LRB1	Early HCC vs. HC	88.38	76.79	(77)
SPRY4-IT1	HCC vs. HC	87.30	50.00	(78)
	HBV-HCC vs. LC+ CHB	43.50	86.70	(78)
ZFAS1	HCC vs. HC	55.70	90.00	(79)
UCA1	HCC vs. CHC +HC	91.40	88.60	(75)
	Early HCC vs. HC	93.10	88.60	(75)
LINC00974	HCC vs. HC	51.10	95.60	(80)
LINC01225	HCC vs. HC	76.10	44.30	(81)
UCA1	HCC vs. HC	92.70	82.10	(82)
	HCC vs. CHC	61	71	(82)
WRAP53	HCC vs. HC	85.40	82.10	(82)
	HCC vs. CHC	85.40	71	(82)
lncRNA-D16366	HCC vs. HC+CH	65.50	84.60	(83)
LINC00161	HCC vs. HC	75.00	73.20	(84)
CRNDE	HCC vs. HC	71.00	87.10	(85)
LINC01419	HCC vs. HC	48.40	96.80	(85)
CRNDE and LINC01419	HCC vs. HC	76.70	90.30	(85)
LRB1+AFP	Early HCC vs. HC	82.83	81.16	(77)
LRB1+DCP	Early HCC vs. HC	87.06	91.56	(77)
LRB1+AFP+DCP	Early HCC vs. HC	88.38	76.79	(77)
SHNG1 and AFP	HBV-HCC vs. LC+ CHB	73.40	86.10	(76)
	HBV-HCC vs. HC	96.40	87	(76)
SPRY4-IT1 and AFP	HCC vs. HC	87.30	65.00	(78)
JPX + AFP	HCC vs. HC	97.10	72.20	(74)
linc00152, UCA1, and AFP	HCC vs. HC+ LC+ CHB	82.9	88.2	(86)
PVT1 and uc002mbe.2	HCC vs. HC	60.56	90.62	(87)
AX800134 and uc001ncr	HBV-HCC vs. HC+ CHB	95.00	88.10	(88)
	Early HBV-HCC vs. HC+ CHB	95.70	88.10	(88)
AFP and UCA1	HCC vs. HC	100	74.20	(75)
UCA1 and c-JUN	HCC vs. HC+ CHC	97.10	80	(75)
	Early HCC vs. HC	100	80	(75)
UCA1 and WRAP53	HCC vs. HC + CHC	95.10	82.10	(82)
AFP, DCP and MALAT1	HCC vs. CH +HC	88.60	75.00	(73)
AFP, UCA1, WRAP53	HCC vs. HC+ CHC	100	62.80	(82)
LOC149086, RP11-160H22.5 and XLOC_014172	HCC vs. HC	85.00	95.00	(89)
CTBP, LAMP2, miR-16-2 and miR-21	HCC vs. HC+ CHC	79.50	100	(90)

*CHC, chronic hepatitis C infection; CHB, chronic HBV hepatitis; HCC, hepatocellular carcinoma; HC, healthy control; CH, chronic hepatitis; HCV-HCC, HCC related with HCV infection; LC, liver cirrhosis; UCA1, Urothelial carcinoma associated-1*.

Li et al. (91) identified several candidate lncRNAs that are abnormally expressed in HCC tumor tissues on the basis of the published literature. Serum samples from HCC cohorts and HCs were then used to evaluate the utility of candidate lncRNAs as HCC biomarkers. The results of this study identified two lncRNAs (HULC and Linc00152) as potential HCC biomarkers. Both HULC and Linc00152 were upregulated in the plasma of patients with HCC, and the expression of HULC and Linc00152 in plasma was positively correlated with the expression in tumor tissue. The AUCs of HULC and Linc00152 to distinguish HCC from HC were 0.78 and 0.85, respectively. The AUC of the combination of HULC and Linc00152 to distinguish HCC from the healthy control group was 0.87, and the addition of AFP improved the AUC to 0.89. Thus, circulating HULC and Linc00152 were shown to be potential noninvasive biomarkers for predicting the diagnosis of early HCC. A study also reported that the diagnostic performance of circulating HULC and Linc00152 was superior to these lncRNAs from HCC tissue (92). Furthermore, as an oncogenic lncRNA, HULC promotes the growth of HCC cells by downregulating the expression of the tumor suppressor gene *p18*, and enhances epithelial-mesenchymal transition (EMT) to promote the hepatocarcinogenesis and metastasis of HCC by sponging miR-200a-3p and upregulating the expression of Zinc finger E-box-binding homeobox 1. HULC can also promote HCC progression by activating acyl-CoA synthetase long chain family member 1 and inhibiting miR-372 and miR-9 expression (93–95). The level of circulating HULC was significantly associated with tumor size, recurrence and Edmondson grades (96). LINC00152 regulated HCC development by sponging miR-215 and miR-193a/b-3p (97, 98). The level of circulating Linc00152 was significantly associated with tumor size and differentiation grade (96). Thus, HULC and Linc00152 can be used as indicators of HCC prognosis. A recently study reported that HULC triggers autophagy by stabilizing silent information regulator 1 protein and attenuated the chemosensitivity of HCC cells. Thus, HULC-targeted therapy enhances the chemosensitivity of HCC cells, and HULC can be used as a target to develop a sensitizing strategy for HCC chemotherapy (99). Different laboratories assessed serum lncRNA–urothelial carcinoma associated-1 (lncRNA-UCA1) expression as a novel biomarker for the diagnosis of HCC (75, 82), and the expression of lncRNA-UCA1 was significantly upregulated in HCC tissues compared to adjacent nontumor tissues and upregulated in the plasma of patients with HCC as compared with controls. There was a strong positive association between the expression of lncRNA-UCA1 in plasma and in HCC tumor tissue. Study reported that the sensitivity of lncRNA-UCA1 to distinguish HCC from HCs was between 91.4 and 92.7%, and the specificity was between 82.1 and 88.6%. In these two studies, the diagnostic performance of lncRNA-UCA1 was better than that of AFP; and the sensitivity and specificity of AFP combined with lncRNA-UCA1 in detecting HCC were 100 and 74.2%, respectively. The sensitivity of AFP combined with lncRNA-UCA1 was increased, and the specificity was decreased. In clinical practice, sensitivity may be more important than specificity in the monitoring of patients with HCC, as delayed diagnosis of HCC may lead to early progression to an advanced stage. The aberrant expression of lncRNA-UCA1 promotes HCC cell proliferation, EMT, and invasion and inhibits cell apoptosis by sponging miR-203 and miR-216b and facilitating the G1/S transition through cyclic-dependent kinase 2, which contributes to the occurrence and progression of HCC (95, 100). Elevated circulating lncRNA-UCA1 levels in HCC patients were related to large tumor sizes, vascular invasion, high tumor grades, recurrence, and Edmondson grades. Moreover, the 5-year overall survival of HCC patients with low circulating lncRNA-UCA1 levels was better than that of patients with high circulating lncRNA-UCA1 levels. The results showed that lncRNA-UCA1 may be a prognostic marker for HCC prognosis (82, 100). It was also reported that the sensitivity and specificity of lncRNA-UCA1 combined with c-JUN in the detection of HCC were 97.1 and 80% in the HC and CH groups, respectively. For early HCC detection, the combination of lncRNA-UCA1 and c-JUN reached 100% sensitivity and 80% specificity, and serum levels of lncRNA-UCA1 and c-JUN may be promising biomarkers in the diagnosis of early HCC (75). Although serum lncRNA-UCA1 and c-JUN were detected in patients with CH, high expression levels of lncRNA-UCA1 and C-JUN in serum samples were present in patients with HCC. The optimal cutoff values for lncRNA-UCA1 or C-JUN could discriminate HCC from CHC groups (75, 82). Studies have shown that the sensitivity and specificity of combined AX800134 and uc001ncr in the early diagnosis of HCC from healthy controls and CHB are 95.7 and 88.1%, respectively (88). The above results emphasize the practicability of early diagnosis of HCC based on lncRNAs. The levels of RP11–160H22.5, XLOC_014172, and LOC149086 were upregulated in the plasma of patients with HCC, and the AUCs of the combination of RP11–160H22.5, XLOC_014172, and LOC149086 to distinguish HCC from the healthy control group were 0.999 and 0.896 in the training set and validation set, respectively (89). Circulating RP11–160H22.5, XLOC_014172, and LOC149086 can be used as potential noninvasive biomarkers for the diagnosis of early HCC. Most of the HCC patients presented markedly decreased levels of these lncRNAs for a month after liver resection; however, some HCC patients showed an increased level after liver resection, which was highly associated with metastasis and poor outcomes. These initial studies are encouraging enough to consider lncRNAs as potential biomarkers for HCC, especially given the lack of efficient serum biomarkers for early HCCs, as lncRNAs were shown to be useful outcome predictors in HCC patients. However, most studies on the diagnosis of HCC based on lncRNAs have included subjects with only one or two etiologies. LncRNAs should also be investigated as markers in the diagnosis of HCC associated with nonalcoholic fatty liver disease (NAFLD), alcohol consumption, HBV infection, or HCV infection in large trials with different groups of patients.

Current studies suggest that many lncRNAs are differentially expressed in HCC and that the aberrant expression of lncRNAs is correlated with HCC progression and may serve as markers for the efficacy of treatment or as therapeutic targets of HCC (95, 101, 102). However, highly sensitive and reliable detection approaches, such as next-generation sequencing and digital PCR, are required for assaying clinical samples due to the low expression levels of lncRNAs in peripheral blood.

## Circular RNAs

CircRNA is a new class of noncoding RNA produced by the “reverse splicing” of protein-coding mRNA or linear noncoding RNA, which is connected by upstream 3′ splicing sites and downstream 5′ splicing sites to form covalently closed continuous circRNA (67). Because of the rapid advances in the development of combined high-throughput sequencing and bioinformatics analysis, many circRNAs have recently been discovered. They are highly stable and conservative, widespread, and diverse in eukaryotic cells, and participate in a variety of physiological and pathological processes. Studies have shown that abnormal circRNA expression has been observed in a variety of tumors, such as colorectal cancer, breast cancer, and HCC. An increasing number of studies have shown that circRNAs regulate the occurrence and development of HCC by targeting different miRNA- and protein-coding genes, such as tumor cell proliferation, epithelial-mesenchymal transformation, apoptosis, autophagy, angiogenesis, and metastasis. Many circRNAs are highly expressed in the blood and are resistant to ribonuclease, with high tissue specificity and developmental stage specificity of HCC, thus circRNAs may represent the promising potential biomarkers for HCC diagnosis (103–109).

Several studies have reported abnormal expression of circ_000244, circ_104075, circ_000520, cSMARCA5, and circ_001565 in the serum/plasma of HCC and clarified the diagnostic performance of these circRNAs in HCC (110–115). Zhang et al. revealed that HCC tissues and serum circ_104075 were highly expressed as compared with the control group. The high expression of serum circ_104075 is specific for HCC, and HCC stage is positively correlated with serum circ_104075 level. The AUC value of circ_104075 that distinguishes HCC from HC is 0.973 (sensitivity: 0.960; specificity: 0.983). Circ_104075 showed better diagnostic performance compared with AFP (AUC value: 0.726), des-gamma-carboxyprothrombin (DCP) (AUC value: 0.771), Lens culinaris agglutinin-reactive AFP (AUC value: 0.766), lncRNA, and miRNA biomarkers such as DANCR (AUC: 0.851), miR-223 (AUC: 0.818), miR-21 (AUC: 0.782), and UCA1 (AUC: 0.735); and study showed that the high serum expression of circ_104075 was specific to HCC, so that the circulating circ_104075 can be used as a promising diagnostic biomarker for the early diagnosis of HCC (114). Result suggests that the level of serum circ_104075 in HCC patients has been found to decrease significantly after curative treatment. Circ_104075 promoted HCC tumorigenesis and development by sponging miR-582, which is an inhibitor of yes-associated protein (YAP), a major downstream effector that contributes to liver tumorigenesis. High serum circ_104075 levels in HCC patients were significantly related to advanced cancer stage (114, 116). Other researchers have identified novel and potential serum circRNA panel HCC biomarkers that reliably detect early HCC patients. Circ_000244, circ_000520, and circ_001565 are highly related to HCC with a high-ranking score. The expression levels of circ_000520 and circ_001565 in HCC patients were significantly lower than those in the HC and chronic hepatitis C (CHC) groups, and the level of circ_000244 was significantly higher than that of the healthy control group and CHC group. The AUC values of circ_000244, circ_000520, and circ_001565 discriminate between HCC and healthy groups were 0.974, 0.943, and 0.839, respectively, which are higher than that of AFP (AUC: 0.726). When combined, the circRNA panel showed remarkably high sensitivity (100%) and specificity (83.3%), indicating that the circRNA panel is also a potential diagnostic early HCC biomarker. A study also showed that serum circ_000520 was an independent prognostic factor of recurrence-free survival (RFS) in HCC patients who underwent operative procedures, chemotherapy, and radiotherapy (113). Recent studies reported (111) that plasma hsa_circ_0001445 levels in HCC patients were significantly lower than those in HCs, patients with LC, and patients with CHB. Patients with LC and CHB have lower plasma hsa_circ_0001445 levels than patients with HC; however, no significant differences were found in plasma hsa_circ_0001445 levels between patients with LC and patients with CHB. Therefore, plasma hsa_circ_0001445 can be used to distinguish patients with HCC from patients with HC, patients with LC or patients with CHB. In addition, the combined diagnostic value of plasma hsa_circ_0001445 and serum AFP was also analyzed. Compared with the use of serum AFP level or plasma hsa_circ_0001445 level alone, the efficacy of combined diagnosis of HCC is higher. No significant correlation was found between circ_0001445 and aminotransferase levels, such as alanine aminotransferase and aspartate aminotransferase. These results indicate that plasma hsa_circ_0001445 can be used as a new diagnostic and monitoring biomarker for HCC. Circ_0001445 can inhibit the proliferation, migration and invasion of HCC cells and induce the apoptosis of HCC cells by sponging miR-17 and miR-181b, which can affect HCC progression (108, 112, 115). Circ_0001445 was significantly related to overall survival and RFS in HCC patients who underwent liver resection. Thus, circ_0001445 may serve as a potential predictive biomarker for HCC (109, 114). In addition to serum circRNA as a diagnostic marker for HCC, the abnormal expression of circRNA in PBMCs of HCC patients was also studied (117), and research showed that the expression of circ_0000798 in PBMCs of HCC patients was significantly upregulated. The AUC value that distinguishes HCC from the HC group is 0.703, and the expression of circRNA is positively correlated with tumor size, indicating the potential role of circ_0000798 as a biomarker for early diagnosis of HCC (117). Previous studies have also shown that the diagnostic performance of plasma hsa_circ_0027089 is comparable to that of AFP which distinguishes HBV-related HCC from HBV-related cirrhosis and healthy participants, plasma hsa_circ_0027089 can serve as a biomarker for diagnosis of HBV-related HCC (118). However, there are not enough preclinical data to demonstrate that circRNAs can serve as targets or therapeutic vectors for cancer treatment. Currently, many studies focus on the diagnosis of HCC; however, circRNAs for the early diagnosis of HCC are mainly related to the use of tissue samples from HCC patients, and studies have shown that circRNAs from HCC tissue samples can be used as effective biomarkers for the early diagnosis of HCC (108, 115, 119, 120). There are few studies on using peripheral blood circRNAs as an early diagnosis tool for HCC, but an increasing number of circRNAs are found to involve in tumor pathophysiology. In future research, the expression of circRNA in noninvasive peripheral blood samples should be detected. These circRNAs show great potential in the diagnosis of HCC (108, 115, 121).

## Exosomes

Exosomes are extracellular vehicles (EVs) with size in 30–200 nm diameter that are secreted by almost all types of cells. They can be found in most body fluids (like serum, plasma, lymph, and saliva, etc.) and have variable components such as encapsulate lipids and multiple types of proteins as well as nucleic acids including mRNA, miRNA, lncRNAs, and DNA detected inside exosomes, which can reflect the status of host cells (122). In the liver, exosomes are mainly released from three types of cells: hepatocytes, immune cells (such as Kupffer cells, natural killer cells, and lymphocytes) and nonparenchymal liver cells (such as hepatic stellate cells). Exosomes are involved in all aspects of intrahepatic cell interactions, and they involve many physiological and pathological processes (123). Accumulating evidence shows that mRNA, microRNA, lncRNA, protein, and circRNA transmitted by exosomes play an important role in the occurrence and progression of HCC (123–125). Tumor cells derive exosomes to communicate with neighboring or distant cells to further promote tumor growth, progression and metastasis (126). Tumor-derived exosomes can enter the circulatory system and be easily detected in the blood (127–132). Recently, an increasing number of studies have suggested that nucleic acids in exosomes are a group of molecules that can serve as HCC biomarkers (132–134). Exosomal circRNAs can distinguish colon cancer patients from HC patients (135), demonstrating their significance as potential noninvasive biomarkers for the diagnosis of tumors. Recently, studies have shown that circRNAs are abundant and stable in HCC cell exosomes (136), and *in vivo* and *in vitro* studies have shown that exosomal circRNAs, such as circPTGR1, Cdr1as, and circDB, were significantly elevated in the serum of HCC patients and promoted HCC cell proliferation and metastasis (124, 136–138). Studies have shown that the expression of exosomal circPTGR1 in the serum of HCC patients is significantly increased and positively correlated with tumor-node-metastasis (TNM) stage as compared with HCs (136). Therefore, exosomal circRNA may be a promising diagnostic biomarker for HCC, but this requires further investigations.

Several studies have shown that exosomal mRNA, microRNA, and lncRNA can be used as biomarkers for HCC screening (23, 128, 133, 139–144). Xu et al. have shown that the exosomal hnRNPH1 mRNA level in HCC patients is significantly higher than those in the LC, CHB, and HC groups, and its expression level is positively correlated with lymph node metastasis and TNM stage. Gender, age, status of HBsAg, and cirrhosis were found to be unrelated to the expression of exosomal hnRNPH1 mRNA. Exosomal hnRNPH1 mRNA showed a sensitivity and specificity of 85.2 and 76.5%, respectively, for distinguishing HCC from CHB, with an AUC of 0.865. The combination of hnRNPH1 mRNA with AFP significantly increased the AUC value. We searched the exosomal hnRNPH1 mRNA in the exoRBase database established by Li et al., and we found that the expression rank of exosomal hnRNPH1 mRNA in HCC was 0–10% (145). Exosomal hnRNPH1 mRNA may be used as an independent marker for early HCC diagnosis. The results also suggested that exosomal hnRNPH1 mRNA is a prognostic indicator for patients with HCC. Elevated levels of exosomal hnRNPH1 mRNA were related to worse overall survival in patients with HCC (139). Another study evaluated exosomal miR-21-5p and miR-92a-3p as biomarkers for the early screening of HCC in patients from Romania (146). This study included 48 patients diagnosed with HCC during screening, who underwent surgical resection or liver transplantation, 38 patients with LC, and 20 HCs. The analysis of the expression profile indicated that miR-21-5p was upregulated and miR-92a-3p was downregulated in plasma-derived exosomes from HCC subjects compared with LC subjects. AUC for HCC diagnosis based on AFP was 0.72. The combination of AFP and exosomal miR-21-5p and miR-92a-3p in a logistic regression equation for early screening of HCC, and the AUC for the combination of AFP and exosomal miR-21-5p and miR-92a-3p was 0.85, significantly better than serum AFP alone. Circulating exosomal miR-21-5p and miR-92a-3p could be served as potential biomarkers for HCC diagnosis in LC patients subjected to screening and surveillance.

The expression levels of lncRNA ENSG00000258332.1 and LINC000635 in serum exosomes of HCC patients were significantly higher than those of patients with CHB. The AUCs of exosomal lncRNA ENSG00000258332.1 and LINC00635 in discriminating HCC from CHB were 0.719 (71.6% sensitivity and 83.4% specificity) and 0.750 (with 76.2% sensitivity and 77.7% specificity), respectively. The AUC for the combination of lncRNAs (ENSG00000258332.1 and LINC00635) and AFP was 0.894 (83.6% sensitivity and 87.7% specificity), and exosomal ENSG00000258332.1 and LINC000635 were positively correlated with lymph node metastasis, TNM stage and overall survival in HCC patients. These data suggest that these lncRNAs could be potential biomarkers for early HCC diagnosis and the prognosis of HCC (141).

Studies have shown that the serum levels of exosomal miR-122 and miR-148a were significantly higher in early HCC than in LC. The AUCs of exosomal miR-122 and miR-148a in discriminating early HCC from LC were 0.816 and 0.891, respectively. The AUC for the combinations of exosomal miR-122, miR-148a, and AFP in discriminating early HCC from LC was 0.947 (87.0% sensitivity and 90.0% specificity). Exosomal miR-122 had the best diagnostic performance, with an AUC of 0.990 (100% sensitivity and 92.0% specificity), discriminating early HCC from CH. However, exosomal miR-122 and miR-148a were not different between the HCC and CH groups; therefore, the combination of these miRNAs and other markers of HCC diagnosis, such as exosomal lncRNAs, could discriminate early HCC from CH (144). A study also showed that the serum exosomal miR-122 expression level was significantly reduced in transarterial chemoembolization (TACE)-treated HCC patients, and the ratio of miR-122 expression before TACE/after TACE was related to disease specific survival (147). Recently, exosome-based HCC therapeutics have been explored in preclinical studies but not in clinical trials. For example, miR-122 was transfected into adipose tissue-derived mesenchymal stem cells (AMSCs), and the AMSCs delivered miR-122 to HCC cells by AMSC-derived exosomes based on the HCC tropism of AMSCs. Exosomal miR-122 enhanced the chemosensitivity of HCC cells to chemotherapeutic agents such as sorafenib by inducing G0/G1 arrest and apoptosis (124). Exosomal miR-125b levels predicted the recurrence of HCC patients who had undergone curative resection with an AUC of 0.739 (83.0% sensitivity and 67.9% specificity), and the exosomal miR-125b levels were significantly associated with tumor number, TNM stage and overall survival (148). Exosomal miRNA signatures or their combination with traditional biomarkers may be used as a suitable peripheral screening tool for HCC. Therefore, exosomes may be effective markers of early HCC and potential therapeutic targets of HCC, which deserve further study.

## Circulating Tumor DNA

In 1948, Mandel et al. (149) first discovered cfDNA in peripheral blood but released cfDNA into the peripheral blood by normal and malignant cells. In 1977, Leon et al. (150) found for the first time that the concentration of cfDNA in the serum and plasma of cancer patients was significantly higher than that of healthy controls. Extensive studies have demonstrated that cfDNAs in cancer patients harbor tumor-specific genetic alterations. Several subsequent studies have shown that cfDNA in cancer patients possesses cancer-associated molecular characteristics and further confirmed that tumor cells can release DNA into peripheral blood (151–155). ctDNA is a DNA fragment containing tumor-specific genetic alterations released directly from living or dead tumor cells in primary or metastatic tumor tissue into the blood (156, 157). ctDNA represents a small fraction of the total cfDNA in cancer patients (158, 159). Therefore, liquid biopsy can analyze blood or other body fluid ctDNA to obtain genetic or epigenetic information, which can be used for tumor screening, diagnosis, prognosis, treatment monitoring or recurrence (159, 160). Liquid biopsy has a deeper understanding of genetic and epigenetic changes in the peripheral blood of HCC patients. Genetic or epigenetic alterations in cfDNA in association with HCC are detectable by liquid biopsy and are reliable indicators of changes occurring in tumor tissues (140, 159). The liquid biopsy detection method can continuously monitor tumor genes and early diagnosis HCC in a noninvasive and precise way. Evidence shows that circulating free DNA molecules in body fluids such as blood and saliva may be used as early biomarkers of HCC (161–165).

Studies have shown that peripheral blood cfDNA levels in HCC patients are correlated with HCC, and total blood cfDNA and ctDNA concentrations in HCC patients are significantly higher than in patients with HC (166, 167). Nonspecific increases in cfDNA and ctDNA concentrations are associated with HCC tumor size, and cfDNA and ctDNA levels may have potential application value in screening HCC (159, 166, 168–172). One study enrolled 72 patients with HCC, 37 patients with CH or LC, and 41 patients with HC. cfDNA levels in patients with HCC were significantly higher than those in patients with HC or benign patients. The cfDNA levels were positively associated with tumor size. The AUC value for distinguishing HCC from the HC group and benign patients was 0.949 and 0.874, respectively. Combined cfDNA and serum AFP revealed an elevated AUC of 0.974 with 95.1% sensitivity and 94.4% specificity in discriminating HCC from controls (166). One study analyzed the levels of cfDNA and AFP in 24 patients with HCC and 62 patients with CHB, and the results suggested that the cfDNA levels were significantly higher in HCC patients than in CHB patients. cfDNA levels were positively associated with AFP. Yan et al. established a mathematical model including serum AFP level, cfDNA level, and age, collectively referred to as the HCC index. The HCC index diagnoses HCC from patients with CHB with an AUC of 0.98, a sensitivity of 87%, and a specificity of 100%. The performance of the HCC index in the diagnosis of HCC is better than that of cfDNA alone or the AFP alone (165). The combination of liquid biopsy-based cfDNA with other proteins or genetic biomarkers is expected to be a clinical tool for the early diagnosis of HCC.

Cancer-specific genetic and epigenetic aberrations in ctDNA are detectable by liquid biopsy as biomarkers for the diagnosis and monitoring of HCC. In general, ctDNA aberrations include single nucleotide mutations (173, 174), DNA copy number variations (CNVs), and changes in methylation status (175, 176). DNA methylation is a well-studied epigenetic modification; and it is an epigenetic regulator of gene expression and regulates gene expression by affecting changes in chromatin structure, DNA stability, and DNA-protein interactions (177). Increased methylation of tumor suppressor genes is an early event in many tumors; therefore, it can usually be detected in the early stage of cancer or precancerous state (161, 178). ctDNA-bearing cancer-specific methylation patterns have been investigated as feasible biomarkers in cancers (161). A meta-analysis showed that the DNA methylation status of certain genes was related to the occurrence and development of HCC, including *p15* and *p16, APC, SPINT2, SFRP1, TFP12, GSTP1*, and *RASSF1A* (179). Multiple studies have shown that the methylation status of several tumor suppressor genes in HCC plasma and tumor tissue is highly consistent (159). Therefore, different methylation status may become a tool for HCC diagnosis. Research by Wong et al. showed that 92% of HCC patients have plasma/serum methylation of *p15* and *p16*, and the combination of these epigenetic markers may prove valuable for noninvasive HCC diagnosis and monitoring (175). Zhang et al. studied the methylation status of *p16, p15*, and *RASSF1A* in the serum of 50 patients with HCC and 50 HCs, and the results showed that the overall accuracy of predicting HCC was 89%, and the sensitivity and specificity were 84 and 94%, respectively. Changes in DNA methylation status may be a valuable biomarker for early detection and monitoring of the clinical course of HCC (180). Huang et al. conducted a comprehensive analysis of the methylation status of 4 genes, including *APC, GSTP1, RASSF1A*, and *SFRP1*. The results showed that the methylation status of 4 genes from the HC group identified an AUC of HCC of 0.933, while the AUC of a single gene was 0.800–0.881. The results showed that the AUC value for the methylation status of 4 genes in discriminating HCC from HCs was 0.933, while the AUC of a single gene was 0.800–0.881 (181). Using cfDNA samples from 1,098 HCC patients and 835 HC patients, Xu et al. identified an HCC-specific methylation marker panel (*BMPR1A, PSD, ARHGAP25, KLF3, PLAC8, ATXN1, Chr 6:170, Chr 6:3, ATAD2, Chr 8:20*). Since the methylation profiles of HCC tumor DNA and matched plasma ctDNA were highly correlated; they constructed a diagnostic prediction model with these methylation profiles. Their study demonstrated that this diagnostic prediction model showed better diagnostic performance in differentiating HCC from HCs (161). These studies have shown that methylation characteristics of HCC can be reliably identified in peripheral blood samples, which may be used as biomarkers for the diagnosis and prognosis of HCC.

In addition to the changes in methylation status, tumor-specific single nucleotide mutations of various genes have been found in the peripheral blood of HCC patients, including *TP53, HCK* and *CTNNB1* (182–185). Six genes (*TP53, CTNNB1, AXIN1, JAK1, EPS15*, and *CACNA2D4*) are considered to harbor the most important mutations in HCC. Studies have shown that regardless of AFP status, the AUC of these six selected mutations genes to diagnose HCC is 0.92 (186). In another study, 34 long-term follow-up HCC patients with peripheral blood mononucleoside mutations, such as *TP53, CTNNB1, PLCB1, PCLO, ROBO1, BAZ2B*, and *TTN*, and HCC clinical processes were analyzed. Studies have shown that plasma samples have a high degree of consistency with single nucleotide mutations in corresponding tumor tissues. Dynamic changes in single nucleotide mutations were well correlated with the patient's tumor burden, ctDNA mutation profiles accurately assessed the patient's tumor burden with high consistency as compared with imaging results, and ctDNA mutation profiles could discover tumor occurrence early in advance of imaging for an average of 4.6 months and showed superior performance as compared with serum biomarkers AFP and DCP (187). Therefore, tumor-specific single nucleotide mutation profiles may be used as biomarkers for the early detection of HCC (188, 189). Studies have also shown that the *TP53* p.R249S mutation is more common in HBV- and aflatoxin-associated HCC than in HCC associated with other etiologies in peripheral blood, and the detection of the *TP53* p.R249S mutation shows promise but may only be highly specific for HCC in certain regions and races. In China, the *TP53* p.R249S mutation detected in HCC cases was higher than that detected in Thailand and Africa (159, 190, 191). Ethnicity and the significant heterogeneity of HCC genetics in association with different etiologies (for instance, alcohol-related liver disease vs. HBV vs. HCV vs. NAFLD) have posed a major challenge to the development of a universal biomarker panel for detecting HCC. Studies have shown that the clinical significance of plasma DNA for copy number aberrations (CNAs) in high-risk populations warrants further investigation (192). For high-risk populations such as LC without any DNA for CNA in plasma, none of them developed HCC during the follow-up period. Plasma DNA for CNA might have future value in screening HCC in high-risk populations. However, CNAs are present in almost all types of cancer (193), which is also a challenge for us to find HCC-specific CNA patterns to diagnose early HCC.

## Conclusions

Although the above described serum genetic markers have better relative sensitivity and specificity, there are still many limitations to be overcome for the diagnosis of early HCC. The main limitation of most studies using nucleic acid molecules as biomarkers of HCC is the limited size of the cohort, and the heterogeneity of HCC should be also considered. A larger patient cohort including patients with different liver disease conditions, such as alcohol-induced HCC, HBV/HCV-associated HCC and HCCs caused by other etiologies, is necessary to achieve a comprehensive analysis, and also the different races can be the basis for the evaluation of the clinical usefulness of the suggested gene biomarkers. Ideal biomarkers not only have sufficient sensitivity and specificity but perhaps more importantly, they must be widely used for monitoring and cost-effectiveness. The cost of separation, processing and amplification of these molecules, as well as quantitative detection and sequencing, might be so high that it would be difficult to apply on a large scale. These small numbers of cohorts can lead to promising results in research, but verification in large cohorts and the establishment of standard cut-off values for screening and diagnostic purposes are still needed. In addition, the establishment of a standardized test is essential for effective screening and diagnosis. Finally, due to the high heterogeneity of HCC, a panel containing multiple genetic and epigenetic alterations as well as protein and metabolite biomarkers could achieve the best diagnostic sensitivity and specificity to distinguish HCC from liver fibrosis or cirrhosis. CancerSEEK analysis detects eight tumor-associated protein biomarkers and mutations in 1933 distinct genomic positions with 98% sensitivity and 99% specificity for HCC detection with an estimated cost of $500 to perform a test (194).

## Author Contributions

TS, LL, and BS conceived of the presented idea. TS, SW, and BS wrote the article, and BS supervised the manuscript. All authors listed have made a substantial, direct and intellectual contribution to the work, and approved it for publication. All authors read and approved the final manuscript.

## Conflict of Interest

The authors declare that the research was conducted in the absence of any commercial or financial relationships that could be construed as a potential conflict of interest.

## Abbreviations

HCC: Hepatocellular carcinoma
AFP: Alpha-fetoprotein
CT: Computed tomography
HBV: Hepatitis B virus
HCV: Hepatitis C virus
LC: Liver cirrhosis
MRI: Magnetic resonance imaging
US: Ultrasound
cfDNA: Cell-free DNA
ctDNA: Circulating tumor DNA
miRNA: MicroRNA
mRNA: Messenger RNA
HCs: Healthy controls
CH: Chronic hepatitis
AUC-ROC: Area under the curve-receiver operating characteristic curve
CHB: Chronic hepatitis B
lncRNA: long noncoding RNA
circRNA: Circular RNA
CNVs: copy number variations
CNAs: copy number aberrations.
